# The IDA-LIKE peptides IDL6 and IDL7 are negative modulators of stress responses in *Arabidopsis thaliana*

**DOI:** 10.1093/jxb/erx168

**Published:** 2017-06-06

**Authors:** Ane Kjersti Vie, Javad Najafi, Per Winge, Ester Cattan, Michael Wrzaczek, Jaakko Kangasjärvi, Gad Miller, Tore Brembu, Atle M Bones

**Affiliations:** 1 Cell, Molecular Biology and Genomics Group, Department of Biology, Norwegian University of Science and Technology, Trondheim, Norway; 2 The Mina and Everard Goodman Faculty of Life Sciences, Bar-Ilan University, Ramat-Gan, Israel; 3 Division of Plant Biology, Department of Biosciences, University of Helsinki, Finland; 4 Distinguished Scientist Fellowship Program, College of Science, King Saud University, Riyadh, Saudi Arabia

**Keywords:** Abiotic stress, Arabidopsis, IDA-LIKE, peptide ligand, ROS, transcriptome, ZAT12

## Abstract

Small signalling peptides have emerged as important cell to cell messengers in plant development and stress responses. However, only a few of the predicted peptides have been functionally characterized. Here, we present functional characterization of two members of the IDA-LIKE (IDL) peptide family in *Arabidopsis thaliana*, IDL6 and IDL7. Localization studies suggest that the peptides require a signal peptide and C-terminal processing to be correctly transported out of the cell. Both *IDL6* and *IDL7* appear to be unstable transcripts under post-transcriptional regulation. Treatment of plants with synthetic IDL6 and IDL7 peptides resulted in down-regulation of a broad range of stress-responsive genes, including early stress-responsive transcripts, dominated by a large group of *ZINC FINGER PROTEIN* (*ZFP*) genes, *WRKY* genes, and genes encoding calcium-dependent proteins. *IDL7* expression was rapidly induced by hydrogen peroxide, and *idl7* and *idl6 idl7* double mutants displayed reduced cell death upon exposure to extracellular reactive oxygen species (ROS). Co-treatment of the bacterial elicitor flg22 with IDL7 peptide attenuated the rapid ROS burst induced by treatment with flg22 alone. Taken together, our results suggest that IDL7, and possibly IDL6, act as negative modulators of stress-induced ROS signalling in Arabidopsis.

## Teaser

The two IDA-LIKE signalling peptides, IDL6 and IDL7, suppress expression of a number of stress-related transcription factors; functional studies suggest a role in modulation of stress-induced ROS signalling.

## Introduction

Plants cannot escape their environment, and are thus exposed to herbivore grazing, pathogen attacks, and other environmental perturbations such as drought, temperature changes, and high salinity. Plants have evolved sophisticated mechanisms in order to meet these challenges. At the cellular level, environmental cues are perceived either directly or indirectly and transduced through a complex signalling network, resulting in an appropriate response by changing the chemical environment of the cells. This transduction typically involves alternations of secondary messengers and signalling molecules such as calcium and phytohormones, but also reactive oxygen species (ROS).

Most types of environmental stresses such as wounding, drought, salinity, heat, cold, and pathogen attack lead to early and rapid accumulation of ROS (Miller *et al.*, 2008; Torres, 2010; Sierla *et al.*, 2013; Gilroy *et al.*, 2014), known as the oxidative burst. Depending on the type of stress and the host involved, the oxidative burst relies on different types of enzymes, including cell wall peroxidases (Bindschedler *et al.*, 2006) and plasma membrane NADPH oxidases (Torres *et al.*, 2002; Miller *et al.*, 2009; Torres, 2010). NADPH oxidases, often referred to as respiratory burst oxidase homologues (Rbohs), are transmembrane proteins responsible for the production of extracellular superoxide (O_2_·^−^) upon pathogen attack (Torres *et al.*, 2002) or abiotic stresses (Kwak *et al.*, 2003; Miller *et al.*, 2009). Both abiotic and biotic stresses trigger a systemic autopropagating wave of ROS, mediated by the NADPH oxidase RBOHD, that travels rapidly in the apoplast from the affected tissue to the entire plant and activates a systemic response to the stress (Miller *et al.*, 2009; Dubiella *et al.*, 2013; Gilroy *et al.*, 2014).

External stress triggers and activates intercellular signalling, essential for cell to cell communication. Since the discovery of systemin in tomato (Pearce *et al.*, 1991), small post-translationally modified peptides have been recognized as important mediators of cell to cell communication (Butenko *et al.*, 2009; Matsubayashi, 2014). They are characterized as small proteins with an N-terminal signal peptide directing the protein to the secretory pathway, a variable region, and a conserved C-terminal part containing the active peptide that after translation undergoes a series of modifications (Matsubayashi, 2014). The translated propeptide is proteolytically processed into the shorter active peptide, which may be further modified (Murphy *et al.*, 2012) before the mature peptide can bind its receptor(s). One well-studied peptide is CLAVATA3 (CLV3) (Clark *et al.*, 1995; Fletcher *et al.*, 1999). Mature CLV3 is modified with two hydroxyprolines; one of these is further modified with three arabinose molecules, enhancing the binding affinity of the peptide for its receptor (Ohyama *et al.*, 2009).

Although several peptides have been linked to growth and development, there are a few studies in Arabidopsis linking signalling peptides to plant stress responses. In Arabidopsis, the first defence-related peptide identified was the plant elicitor peptide (Pep) AtPep1, derived from the precursor peptide PROPEP1 (Huffaker *et al.*, 2006). AtPep1 binds the two leucine repeat-rich receptor-like kinases (LRR-RLKs) PEPR1 and PEPR2 (Yamaguchi *et al.*, 2006, 2010), and acts as a damage-associated molecular pattern (DAMP) promoting the expression of pathogen defence genes such as PLANT DEFENSIN 1.2 (PDF1.2) and PATHOGEN-RELATED 1 (PR1), thus amplifying the defence response upon pathogen attack (Yamaguchi *et al.*, 2006, 2010). The growth-promoting phytosulfokine (PSK) peptide has also been linked to plant defence. It has been shown that PSK through its receptor PSK RECEPTOR 1 (PSKR1) attenuates pattern-trigged immunity in Arabidopsis, suggesting that PSK/PSKR1 is part of a mechanism that controls the allocation of resources between growth and immunity (Igarashi *et al.*, 2012). Knockout plants of the C-TERMINALLY ENCODED PEPTIDE family member CEP3 show increased root and shoot growth compared with the wild type under several abiotic stress conditions, suggesting a role for CEP3 as a negative regulator of growth in response to changing environmental conditions (Delay *et al.*, 2013). Overexpression of oxidative stress-induced peptide OSIP108 resulted in increased tolerance to the ROS inducer paraquat (De Coninck *et al.*, 2013), and proteolytic processing of apoplastic GRIM REAPER by METACASPASE-9 produced an 11 amino acid ligand for its receptor PRK5 that is active in RbohD-dependent cell death (Wrzaczek *et al.*, 2015). The PAMP-induced secreted peptide 1 (PIP1) and 2 (PIP2) have been shown to be induced by a variety of pathogens and elicitors, and PIP1 was found to amplify the immune response in a PEP1-like fashion through the LRR-RLK RLK7 (Hou *et al.*, 2014). Finally, the cysteine-rich peptide AtCAPE1 confers salt sensitivity, in line with a negative regulatory role in salt stress tolerance (Chien *et al.*, 2015).

We have recently performed a genome-wide screen for genes encoding small signalling peptides with similarity to INFLORESCENCE DEFICIENT IN ABSCISSION (IDA) in *Arabidopsis thaliana* (Vie *et al.*, 2015). *IDA* belongs to a family of nine genes, where *IDA-LIKE 1* (*IDL1*) to *IDL5* have been postulated to play roles during plant development (Butenko *et al.*, 2003; Stenvik *et al.*, 2008). Two newly discovered members of this subfamily, *IDL6* and *IDL7*, are closely related, and expression analysis revealed that these two genes are induced by various stress treatments (Vie *et al.*, 2015). In this study, we aimed to characterize the putative ligands IDL6 and IDL7 and their roles in modulating stress responses.

## Materials and methods

### Plant material

Seeds of the *Arabidopsis thaliana* ecotype Col-0 (N1092), *idl6-1* (SALK_074245), *idl6-2* (SALK_126026) (Alonso *et al.*, 2003), and *idl7* (WDL293-296; Woody *et al.*, 2007) mutants were obtained from the European Arabidopsis Stock Centre (NASC, Nottingham, UK), and T-DNA insertions were confirmed by PCR using gene- and T-DNA-specific primers (see Supplementary Table S1 at *JXB* online). Verified homozygous lines were back-crossed to Col-0 wild type to ensure single knockout lines. Double knockout lines were obtained by crossing *idl7* (pollen) to *idl6-1* (mother plant) and *idl6-2* (pollen) to *idl7* (mother plant). Double homozygous lines were verified by PCR using gene- and T-DNA-specific primers (Supplementary Table S1).

*IDL6* and *IDL7* promoter:β-glucuronidase (GUS) fusions were generated using Gateway technology (Invitrogen). The intergenic regions upstream of *IDL6* (869 bp) and *IDL7* (1306 bp) were amplified from genomic DNA fromthe Col-0 ecotype using the primers pIDL6attB1 and pIDL6attB2 for the *IDL6* promoter region, and pIDL7attB1 and pIDL7attB2 for the *IDL7* promoter region (Supplementary Table S1). The fragments were cloned upstream of the *GUS* gene in the destination vector pMDC163 (Curtis and Grossniklaus, 2003) via the pDONR/ZEO vector (Invitrogen). Complementation lines were generated by amplifying the region surrounding *IDL6* (860 bp upstream and 142 bp downstream coding sequence) and *IDL7* (1306 bp upstream and 336 bp downstream coding sequence) from genomic DNA from the Col-0 ecotype using the primers pIDL6attB1 and IDL6comp attB2 for *IDL6* and pIDL7atttB1 and IDL7comp attB2 for *IDL7* (Supplementary Table S1). The fragments were cloned into the destination vector pMDC99 (Curtis and Grossniklaus, 2003). The constructs were introduced into *Agrobacterium tumefaciens* strain C58C1 pGV2260 and transformed into Arabidopsis Col-0 ecotype using the ‘floral dip’ method (Clough and Bent, 1998). Positive transformants were selected on half-strength solid Murashige and Skoog (MS) medium containing the T-DNA-specific selection marker hygromycin (20 µg ml^–1^).

### Subcellular localization

Full-length (*IDL7*_*FL*_) or mutated [*IDL7*_*∆SP*_, IDL7 lacking the predicted signal peptide (SP); *IDL7*_*∆SP∆C*_, IDL7 lacking both the SP and the four last C-terminal amino acids; *IDL7*_*∆C*_, full-length IDL7 lacking the four last C-terminal amino acids] coding sequences of *IDL7* were amplified from Col-0 ecotype genomic DNA using the primers SPIDL7attB1, IDL7DSPattB1, IDL7USattB2, and ILD7EPIPattB2 (Supplementary Table S1), and cloned into the destination vector pEG103 (Earley *et al.*, 2006) by Gateway technology (Invitrogen). The vectors were introduced into *A. tumefaciens* strain C58C1 pGV2260, and transformed cultures [optical density at 600 nm (OD_600_)=0.05, 10 mM MES, 10 mM MgCl_2_, and 100 µM acetosyringone] were used for infiltration of leaves of 3- to 4-week-old *Nicotiana benthamiana* plants grown on soil under a 16 h photoperiod (70 µmol m^−2^ s^−1^) at 22 °C as described (Sparkes *et al.*, 2006). Infiltrated leaves were investigated 2–4 d post-infection using a Leica TCS SP5 confocal laser scanning microscope (Leica Microsystems, Wetzlar, Germany) with a ×50 water immersion objective. Green fluorescent protein (GFP) was excited using an argon laser at 488 nm, and emission was detected at 495–550 nm.

### Histochemical GUS assays

Histochemical GUS assay was performed as described by Butenko *et al.* (2003).

### RNA decay analysis

RNA decay assays were carried out as described (Zhang *et al.*, 2010). Four-day-old seedlings were pre-treated in incubation buffer (1 mM PIPES, pH 6.25, 1 mM sodium citrate, 1 mM KCI, 15 mM sucrose), and then supplied with cordycepin (Sigma-Aldrich, St Louis, MO, USA) to a final concentration of 1 mM. Samples were collected before (0 min) and 30 min and 60 min after cordycepin addition. Tissues for each time point were flash-frozen in liquid nitrogen and stored at –80 °C.

### Peptide treatments

Synthetic peptides (Biomatik, Canada) used in this study are listed in Table 1. Seeds of the Col-0 ecotype were surface-sterilized and sown out on half-strength MS plates at a density of 20 seeds per Petri dish (14 cm diameter), and stratified for 3 d at 4 °C. Plates were grown under a 16 h photoperiod (70 µmol m^−2^ s^−1^) at 22 °C for 2 weeks. Seedlings were sprayed with an aqueous peptide solution (100 nM) supplemented with 0.02% Silwet L-77 (Lehle Seeds, Round Rock, TX, USA) and vacuum infiltrated at 20 inches Hg for 1 min. Whole rosettes were collected 1, 2, and 3 h after treatment, snap-frozen in liquid nitrogen, and stored at –80 °C.

**Table 1.** Synthetic peptides used in this study

Name	Position	Sequence
IDL6-EPIP	75–98	FGSLVLNALP^10^KGSVPASGPS^20^KRIN
IDL7-EPIP	70–93	FGSLVLNALP^10^KGSRPGSGPS^20^KKTN
IDL7-PIP	82–93	SRPGSGPSKK^10^TN
IDL7-PIPo	82–93	SRPGSGhypSKK^10^TN^*a*^
MOCKIDL7	75–98	LSPGKNLSAP^10^GRVGSNPFTK^20^LRGS

^*a*^hyp, hydroxyproline

### Expression analyses

RNA isolation, cDNA synthesis, real-time quantitative reverse transcription–PCR (qRT–PCR), microarray experiments, statistical analysis, and Gene Ontology (GO) analysis were performed as described in Vie *et al.* (2015). In brief, RNA was extracted from 100 mg of frozen plant tissue each from four biological replicas using the Spectrum Plant Total RNA kit (Sigma-Aldrich). cDNA synthesis was performed on 1 µg of total RNA using the QuantiTect Reverse Transcription Kit (Qiagen, Hilden, Germany), following the supplier’s instructions. qRT–PCR was performed on a LightCycler 480 using the LightCycler 480 SYBR Green I Master kit (Roche Applied Science, Mannheim, Germany), with PCR parameters as recommended by the supplier. PCR efficiencies and C_t_ values were calculated by linear regression using the LinRegPCR software (Ramakers *et al.*, 2003; Ruijter *et al.*, 2009), and mean PCR efficiency was calculated for each pair of primers. C_t_ values and PCR efficiencies were then imported into the REST 2008 software (Pfaffl *et al.*, 2002) to calculate the statistical significance of differences in expression levels upon various treatments. Primers used are listed in Supplementary Table S1. Genome-wide expression analysis was performed using the Arabidopsis (V4) Gene Expression Microarray 4 × 44K (Agilent Technology, USA) as described in the supplier’s manual. The data were analysed using the limma package (Smyth, 2004) and the R statistical data analysis program package (R 2.10.1). The Benjamini and Hochberg method to control the false discovery rate was used to identify differentially regulated genes (Benjamini and Hochberg, 1995). Genes with an adjusted *P*-value <0.05 were regarded as significantly differentially expressed. The microarray study is MIAME compliant. Raw data have been deposited in GEO (accession GSE77467).

### Luciferase assay

Three-week-old transgenic plants containing the *ZAT12::luc* reporter system (Miller *et al.*, 2009) were grown under a 16 h photoperiod regime and sprayed with 1 mM luciferin the evening prior to the experiment. The next day, plants were uniformly sprayed with luciferin supplemented with each of the three peptides (100 nM). At 65 min after treatment, the plants were injured by pricking one leaf. In total three plants per peptide were treated in each live imaging experiment. Luminescence imaging and measuring was conducted using the NightOWL *in vivo* imaging system (Berthold Technologies, Germany).

### flg22 and H_2_O_2_ treatment

Seeds of the Col-0 ecotype were surface-sterilized and sown out on half-strength MS plates at a density of 20 seeds per Petri dish (14 cm diameter), and stratified for 3 d at 4 °C. Plates were then grown under a 16 h photoperiod (70 µmol m^−2^ s^−1^) at 22 °C for 2 weeks. Seedlings were sprayed with either flg22 (100 nM) or H_2_O_2_ (20 mM) in deionized (DI) water containing 0.02% Silwet L-77 and vacuum infiltrated at 20 inches Hg for 1 min. As control, plants were treated with DI water supplemented with 0.02% Silwet-L77 and vacuum infiltrated at 20 inches Hg for 1 min. Whole rosettes were harvested from four biological replicas at 5, 10, 15, 30, and 60 min after treatment, snap-frozen in liquid nitrogen, and stored at −80 °C.

### Seedling pathogen assay

The protocol was modified from Lee *et al.* (2011): surface-sterilized seeds were sown in 24-well plates containing 2 ml of liquid half-strength MS, with a density of three seedlings per well, and stratified for 3 d at 4 °C. Plants were grown in a growth chamber (VB1514, Vötsch Industrietechnik, Balingen, Germany) under a 16 h photoperiod (70 µmol m^−2^ s^−1^) at 22 °C. *Pseudomonas syringae* pv. *tomato* DC3000 and *P. syringae* pv. *tomato AvrRPM1* cultures were grown overnight in liquid King’s B medium supplemented with the appropriate antibiotics (50 µg ml^−1^ rifampicin for the DC3000 strain, 50 µg ml^−1^ rifampicin, and 50 µg ml^−1^ tetracycline for the AvrRPM1 strain) in a shaker at 28 °C. The cultures were washed twice with 10 mM MgCl_2_ and diluted to OD_600_=0.002 (~2.5 × 10^6^ CFU ml^−1^). Seven-day-old seedlings were transferred to new 24-well plates containing fresh half-strength MS without sucrose, and 100 µl of diluted bacterial culture was added. For measurements of bacterial growth, co-cultivated seedlings were washed with 70% ethanol for 10 s and rinsed in water. Three seedlings were put into eppendorf tubes containing 100 µl of 10 mM MgCl_2_ and ground with a pestle. A 10 µl aliquot of serial dilutions was plated out on LA medium containing the appropriate antibiotics and incubated for 2 d at 28 °C. All pathogen assays were repeated at least three times with similar results.

### Stress assays

Seeds of wild-type Col-0 and loss-of-function mutants of *IDL6* and *IDL7* were surface-sterilized, placed in rows on square half-strength MS plates (control) and half-strength MS with added NaCl (100 mM), mannitol (300 mM), or flg22 (100 nM), and stratified for 3 d at 4 °C. Plates were then placed vertically in a growth chamber (VB1514, Vötsch Industrietechnik) under a 16 h photopheriod (70 µmol m^−2^ s^−1^) at 22 °C. Root lengths were scored 1 week after seed plating for NaCl and mannitol treatments, and at 10 d for flg22 treatments. The X/XO assay was performed as described (Overmyer *et al.*, 2000). All stress assays were repeated at least three times with similar results.

### ROS assay

ROS measurements on Arabidopsis leaf disks were performed as described by Bisceglia *et al.* (2015). Briefly, rosette leaves from 4-week-old plants grown at 22 °C, with a 12 h light/12 h dark photoperiod were detached and rinsed in DI water. Leaf disks were made using a puncher, incubated for 2 h in DI water, and transferred to a 96-well plate containing DI water. The plate with leaf disks was incubated in darkness overnight. DI water was then replaced with luminol solution (30 μg luminol ml^–1^, 20 μg of horseradish peroxidase ml^–1^). Water or 100 nM peptide was added and ROS production was detected as luminescence was measured in a plate reader (Synergy H1; BioTek, Winooski, VT, USA) for 40 min.

## Results

### IDL7 is processed C-terminally prior to export from the cell

**Fig. 1.**
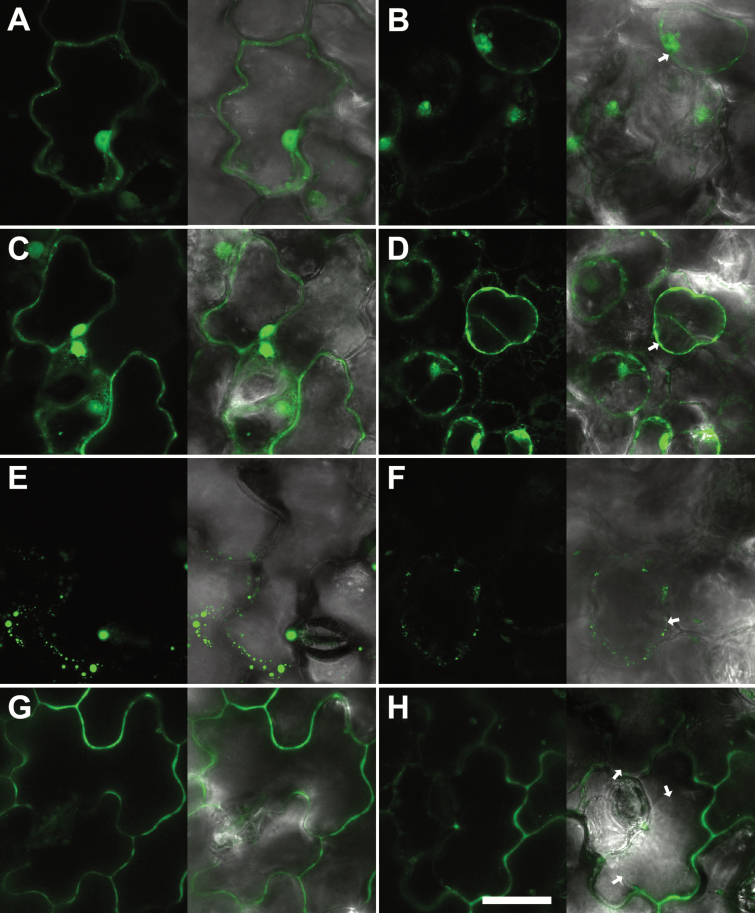
IDL7 is localized to the apoplast. C-terminal GFP tags were cloned in-frame with IDL7, expressed in *N. benthamiana* epidermal cells under control of the 35S promoter, and analyzed using confocal laser scanning microscopy. The GFP control was localized in the cytosol and the nucleus (A), confirmed by plasmolysis (1 M NaCl, 30 min) (B). (C) IDL7_∆SP_–GFP was localized in the cytosol, confirmed by plasmolysis (1 M NaCl, 30 min) (D). (E) IDL7_FL_–GFP was found clogged in vesicular compartments, confirmed by plasmolysis (1 M NaCl, 30 min) (F). (G) IDL7_∆C_–GFP was localized in the apoplastic space, confirmed by plasmolysis (1 M NaCl, 30 min) (H). Arrows indicate plasmolysed plasma membrane. Scale bar=20 ìm.

One of the hallmarks of the IDA family proteins is the presence of an N-terminal SP for the secretory pathway. IDA has previously been shown to be localized to the apoplast (Butenko *et al.*, 2003). To investigate whether IDL7 is secreted out of the cell, we created constructs containing translational fusions between *GFP* and full-length *IDL7* cDNA (IDL7_FL_, corresponding to amino acids 1–97) or IDL7 without the predicted N-terminal SP (*IDL7*_∆*SP*_, corresponding to amino acids 22–97), as shown in Supplementary Fig. S1. The protein fusions were transiently expressed in *N. benthamiana* leaves, and the subcellular localization was examined by confocal microscopy. All constructs produced a strong fluorescence signal; the GFP control was located in the cytosol and nucleus (Fig. 1A). IDL7_∆SP_::GFP was, as expected, located in the cytosol, and could also be found in the nucleus (Fig. 1C), resembling the GFP control. This localization was confirmed by plasmolysis with 1 M NaCl (GFP control, Fig. 1B; and IDL7_∆SP_::GFP, Fig. 1D). IDL7_FL_::GFP localization appeared to be intracellular (Fig. 1E), with the formation of fluorescent aggregates, in addition to a weak extracellular localization. Plasmolysis verified the intracellular localization (Fig. 1F).

It has been suggested that small signalling peptides might be processed C-terminally as well as N-terminally (Matsubayashi, 2014). The fluorescent aggregates seen in *IDL7*_*FL*_*::GFP*-transformed cells might thus result from proteolytic cleavage of the IDL7 C-terminal end fused with GFP. We therefore generated a new IDL7–GFP fusion without the four C-terminal amino acids of IDL7, corresponding to the C-terminal end of the IDA EPIP (extended PIP) peptide (Stenvik *et al.*, 2008; Supplementary Fig. S1). In cells expressing IDL7_∆C_::GFP, however, the fluorescent signal was detected at the surface of the cell (Fig. 1G). In plasmolysed cells expressing IDL7_∆C_::GFP, fluorescence was observed in the extracellular space, between the detached plasma membrane and the cell wall (Fig. 1H). Altogether, these results show that IDL7 is indeed exported from the cell, and that IDL7 is processed C-terminally as well as N-terminally during maturation.

### Rapid turnover of *IDL6* and *IDL7* mRNA

**Fig. 2.**
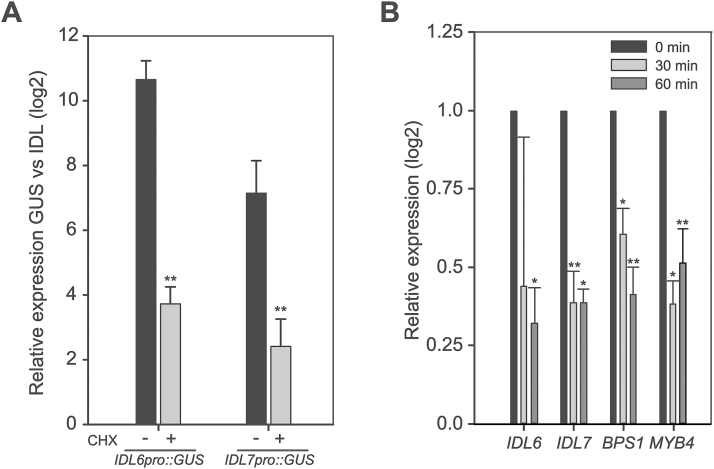
Rapid turnover of *IDL6* and *IDL7* mRNA. (A) Ten-day-old reporter lines (*n*=3) expressing *GUS* under control of the *IDL6* and *IDL7* promoters were analysed for *GUS* and *IDL6/IDL7* expression through qRT–PCR with (two independent lines) or without (four independent lines) CHX treatment (10 µg ml^–1^, 3 h). The mean and SD of the expression ratio (log_2_) between *GUS* and *IDL6*/*IDL7* for each construct is shown. (B) mRNA decay analysis showing the relative gene expression level of *IDL6* and *IDL7* at 30 min and 60 min after adding of cordycepin (1 mM) to the media. *BPS1* and *MYB4* were included as controls (*n*=4). Statistical differences (REST analysis: *P-value <0.05; **P-value <0.01) between the time point samples and control are indicated. Error bars indicate SDs.

*IDL6*_*pro*_ *:GUS* and *IDL7*_*pro*_*:GUS* lines were generated to investigate the expression pattern of these genes. However, the expression patterns indicated by GUS staining did not match the expression pattern found by qRT–PCR (Vie *et al.*, 2015); instead, *GUS* appeared to be constitutively expressed at high levels in most tissues (Supplementary Fig. S2). This, together with the elevated expression level of *IDL6* and *IDL7* observed upon cycloheximide (CHX) treatment (Vie *et al.*, 2015), led us to perform a comparison of the expression levels of *IDL6/IDL7* and *GUS* in four independent lines each of *IDL6*_*pro*_*:GUS* and *IDL7*_*pro*_*:GUS*. *GUS* expression in *IDL6*_*pro*_*:GUS* and *IDL7*_*pro*_*:GUS* lines was 1000-fold and 200-fold higher than the *IDL6* and *IDL7* expression levels, respectively (Fig. 2A). In comparison, the *GUS* expression after CHX treatment in *IDL6*_*pro*_*:GUS* lines was only up-regulated 13-fold compared with *IDL6*, and 5-fold up-regulated in the *IDL7*_*pro*_*:GUS* lines compared with *IDL7* (Fig. 2A; Supplementary Fig. S2). One consequence of the inhibitory effect of CHX on translation is that mRNA molecules become trapped on polysomes, thereby preventing mRNA degradation (Cochran *et al.*, 1983; Edwards and Mahadevan, 1992). Therefore, we measured mRNA decay on *IDL6* and *IDL7* at early growth stages using the transcription inhibitor cordycepin (Johnson *et al.*, 2000). mRNA from both *IDL6* and *IDL7* showed a rapid decay shortly after cordycepin treatment (Fig. 2B), confirming the GUS assay results. Taken together, the results suggest that IDL6 and IDL7 are under strong regulation, at both transcriptional and post-transcriptional level.

### IDL7 peptide down-regulates many stress-responsive genes

**Fig. 3.**
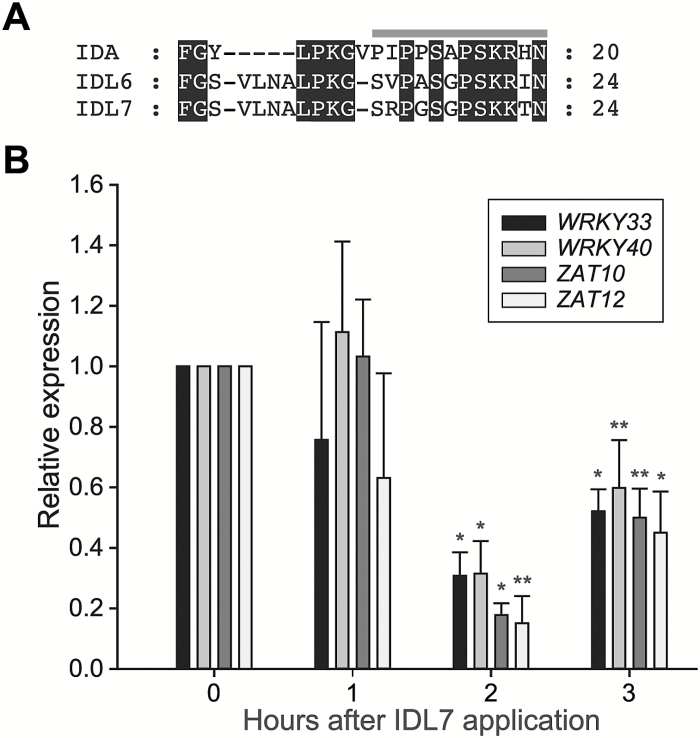
IDL7 negatively affects the expression of stress-related genes. (A) Alignment of the EPIP peptides from IDA, IDL6, and IDL7. The grey bar above the alignment indicates the PIP peptide. (B) Effect of the IDL7-EPIP peptide on gene expression of the transcription factor genes *WRKY33*, *WRKY40*, *ZAT10*, and *ZAT12* over time. Statistical differences (REST analysis: *P-value <0.05; **P-value <0.01) between samples treated with MOCK_IDL7_ and IDL7 peptide (100 nM) are indicated (*n*=3). Error bars indicate SDs.

The bioactive part of the IDA peptide has been identified to be within the C-terminal part, between amino acids 50 and 69. This 20 amino acid peptide, named IDA EPIP, is sufficient to rescue the floral abscission-deficient phenotype of the *ida* mutant *in vitro* (Stenvik *et al.*, 2008). The corresponding EPIP motifs from IDL6 and IDL7, while somewhat longer (24 amino acids), are similar to IDA in 12 positions (Fig. 3A). Peptides corresponding to the EPIP motifs of IDL6 and IDL7 were synthesized and tested for bioactivity in a pilot experiment by spraying wild-type plants with a 10 µM peptide solution. No phenotypes were observed on the plants after treatment; however, a microarray analysis of plants 3 h after treatment suggested that both peptides induced significant transcriptome changes (results not shown). Among the most down-regulated genes were the stress-related transcription factor genes *WRKY33*, *WRKY40*, *ZAT10*, and *ZAT12.* These genes were selected for a closer analysis of the timing of the response to IDL7 peptide. The peptide concentration was reduced to 100 nM, which is comparable with other studies (Yamaguchi *et al.*, 2006; Delay *et al.*, 2013; Chien *et al.*, 2015; Wrzaczek *et al.*, 2015). qRT–PCR showed that the strongest transcriptional response was observed 2 h after application of peptides (Fig. 3B). This time point was chosen for global transcription profiling.

Global transcriptome profiles were obtained from Arabidopsis seedlings following 2 h treatments with 100 nM IDL6-EPIP or IDL7-EPIP. Treatment with the IDL7 EPIP peptide resulted in significant changes in the transcriptome compared with mock peptide-treated plants; in total, 939 genes were found to be significantly regulated (log_2_>0.5 and log_2_< –0.5; *P*<0.05; Supplementary Dataset S1). Table 2 presents the most highly regulated genes. Interestingly, a majority of the genes (73%) were down-regulated. Treatment with the IDL6-EPIP peptide resulted in a much weaker response, with only 42 significantly regulated genes (log_2_>0.5 and log_2_< –0.5; *P*<0.05; Supplementary Dataset S2). When using less strict statistical criteria for IDL6-regulated genes (log_2_>0.5 and log_2_< –0.5; *P*<0.1), the number of significantly regulated genes was still lower (493 genes; 270 up-regulated, 298 down-regulated) compared with IDL7-treated plants. More than half of the genes regulated by IDL6-EPIP treatment (using the less stringent statistical criteria) were also regulated by IDL7-EPIP treatment (Supplementary Fig. S3). This suggests that despite the strong homology of IDL6 and IDL7 peptide, the effect of IDL7-EPIP on the transcriptome is much stronger at the given developmental stage and environmental conditions than IDL6-EPIP.

**Table 2.** The most up- and down-regulated genes 2 h after treatment with IDL7-EPIP peptide (*P*<0.05) Genes with expression ratios of log_2_>1.0 or log_2_< –1.5 are listed

Description	Locus	Log _ 2 _ fold change	Variance	Adjusted *P*-value
ASN1 (GLUTAMINE-DEPENDENT ASPARAGINE SYNTHASE 1)	At3g47340	1.519	0.252	0.013
SPla/RYanodine receptor (SPRY) domain-containing protein^*a*^	At4g06536	1.279	0.125	0.010
ADS1 (DELTA 9 DESATURASE 1)^*b*^	At1g06080	1.218	0.218	0.033
SAUR26 (SMALL AUXIN UP RNA 26)^*b*^	At3g03850	1.200	0.022	0.010
ATMAGL4 (MONOACYLGLYCEROL LIPASE 4)^*b*^	At1g73480	1.151	0.231	0.023
SAUR29 (SMALL AUXIN UP RNA 29)^*a*,*b*^	At3g03820	1.127	0.077	0.008
Unknown protein^*a*^	At4g33467	1.111	0.457	0.015
HMT3 (HOMOCYSTEINE S-METHYLTRANSFERASE 3)^*a*^	At3g22740	1.097	0.084	0.017
A_thal_3526 family protein^*a*,*b*^	At3g55240	1.030	0.034	0.013
P5CS1 (DELTA1-PYRROLINE-5-CARBOXYLATE SYNTHASE 1)^*a*^	At2g39800	1.023	0.057	0.013
IAA29 (INDOLEACETIC ACID-INDUCED PROTEIN 29)^*b*^	At4g32280	1.019	0.067	0.010
CYP96A12 (Cytochrome P450 96A12)^*a*^	At4g39510	1.011	0.110	0.016
DUF946 family protein	At2g44230	1.009	0.086	0.020
ABC transporter-related protein	At3g21080	–1.507	0.341	0.012
Protein phosphatase AP2C2^*a*^	At1g07160	–1.523	0.280	0.021
ALAAT2 (ALANINE AMINOTRANSFERASE 2)	At1g72330	–1.532	0.252	0.023
Unknown protein^*a*^	At2g15020	–1.537	0.028	0.008
ST (steroid sulphotransferase)	At2g03760	–1.558	0.637	0.040
DIC2 (Mitochondrial Dicarboxylate Carrier Protein 2)^*a*^	At4g24570	–1.560	0.004	0.008
Glycine-rich protein^*a*^	At1g07135	–1.563	0.014	0.008
Unknown protein	At5g14730	–1.571	0.244	0.017
Unknown protein^*a*^	At1g25400	–1.582	0.440	0.012
ZAT11 (ZINC FINGER OF ARABIDOPSIS THALIANA 11)^*a*^	At2g37430	–1.584	0.275	0.012
UGT73C3 (UDP-GLUCOSYL TRANSFERASE 73C3)	At2g36780	–1.605	0.375	0.029
ZAT8 (ZINC FINGER OF ARABIDOPSIS THALIANA 8)	At3g46080	–1.616	0.607	0.025
Unknown protein	At4g08555	–1.636	0.930	0.036
VQ12 VQ motif-containing protein	At2g22880	–1.643	0.428	0.021
UGT73C6 (UDP-GLUCOSYL TRANSFERASE 73C6)	At2g36790	–1.675	0.535	0.018
2-Oxoglutarate-dependent dioxygenase like protein	At5g43450	–1.676	0.313	0.021
Bifunctional inhibitor/lipid-transfer protein^*a*^	At1g62500	–1.683	0.790	0.018
HSP23.5-M (23.5 kDa heat shock protein, mitochondrial)	At5g51440	–1.701	0.323	0.023
Unknown protein	At5g24640	–1.715	0.811	0.039
Unknown protein	At2g41730	–1.719	0.592	0.022
ZAT7 (ZINC FINGER OF ARABIDOPSIS THALIANA 7)^*a*^	At3g46090	–1.774	0.263	0.013
WRKY40 (WRKY TRANSCRIPTION FACTOR 40)^*a*^	At1g80840	–1.798	0.178	0.012
ZAT6 (ZINC FINGER OF ARABIDOPSIS THALIANA 6)^*a*,*b*^	At5g04340	–1.809	0.114	0.011
Chaperone DnaJ-domain superfamily protein^a^	At1g72416	–1.840	0.226	0.014
WRKY33 (WRKY TRANSCRIPTION FACTOR 33)^*a*^	At2g38470	–1.842	0.204	0.008
PIP1 (PAMP-INDUCED SECRETED PEPTIDE 1)^*a*^	At4g28460	–1.844	0.733	0.040
Acyl-CoA N-acetyltransferase^*a*^	At2g32020	–1.855	0.867	0.047
Ethylene-responsive transcription factor ERF105^*a*^	At5g51190	1.856	0.219	0.008
HSP15.4 (Heat shock protein class V 15.4)^*a*^	At4g21870	–1.913	0.044	0.008
AGC2-1/OXI1 (OXIDATIVE SIGNAL-INDUCIBLE 1)^*a*^	At3g25250	–1.930	0.509	0.028
Unknown protein	At4g12735	–1.979	0.720	0.029
Eukaryotic aspartyl protease family protein	At5g48430	–1.984	0.020	0.008
CYP81D8 (Cytochrome P450 81D8)	At4g37370	–1.989	0.796	0.026
AtOM66 (Outer mitochondrial membrane protein of 66 kDa)	At3g50930	–2.102	0.760	0.031
ATPP2-A5 (PHLOEM PROTEIN 2-LIKE A5)^*a*^	At1g65390	–2.120	0.127	0.001
COG4129-domain protein^*a*^	At3g09450	–2.136	0.099	0.008
UGT74E2 (UDP-GLUCOSYL TRANSFERASE 74E2)	At1g05680	–2.151	1.090	0.048
DUF295-domain protein	At5g55150	–2.202	1.096	0.043
ZAT10/STZ (SALT-TOLERANCE ZINC FINGER)^*a*^	At1g27730	–2.215	0.137	0.008
ZAT12 /RHL41 (RESPONSIVE TO HIGH LIGHT 41)	At5g59820	–2.359	0.619	0.013
CML38 (CALMODULIN-LIKE PROTEIN 38)^*a*^	At1g76650	–2.456	0.121	0.008

^*a*^Significantly regulated 2 h after treatment with IDL6 peptide (*P*<0.1).

^*b*^Identified as PIF-regulated PIF4 target gene (Oh *et al.*, 2012).

**Fig. 4.**
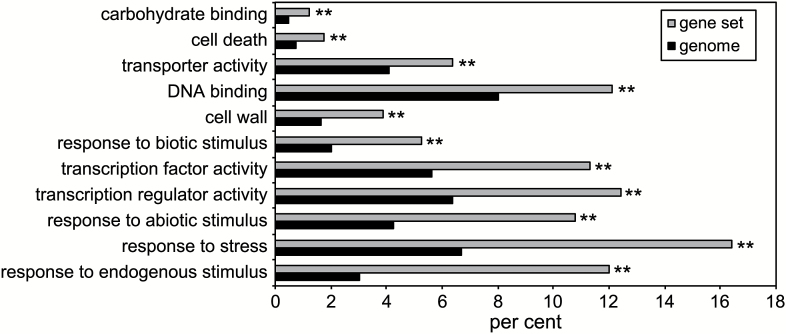
GO enrichment analysis of significantly down-regulated (*P*-value <0.05) genes in 2-week-old seedlings 2 h after treatment with 100 nM IDL7-EPIP peptide. Control seedlings were treated with 100 nM MOCK_IDL7_ peptide. The 10 terms most significantly enriched in the gene set are listed from top to bottom. Bars show the frequency of each GO term in the IDL7-responsive gene set and the genome. **P-value <0.01.

To elucidate a possible function of the IDL7-EPIP peptide, the dataset was analysed for over-representation of Gene Ontology (GO) terms (Ashburner *et al.*, 2000) on genes with log_2_< –0.5; *P*<0.05. These results show a clear over-representation of plant stress-related classes, especially the classes for response to stress, response to endogenous stimulus, responses to abiotic stimulus, and gene regulation (Fig. 4; Supplementary Dataset S3).

The most prominent gene families in the dataset included WRKY transcription factors, ZINC FINGER PROTEINS (ZFP), MYB transcription factors, and ethylene-responsive transcription factors (Table 2; Supplementary Dataset S1). We found 11 significantly down-regulated *WRKY* genes after treatment with the IDL7 peptide; all of them are associated with stress responses. Nine out of 20 genes encoding ZFPs belonging to the C2H2 subfamily C1-2i, all associated with abiotic stress responses, were down-regulated by IDL7. Many other genes associated with stress adaptation or defence were down-regulated, including a large group encoding Ca^2+^-binding proteins, genes encoding heat shock proteins, cytochrome P450-encoding genes, and genes encoding receptor proteins (Table 2; Supplementary Dataset S1).

Genes up-regulated by IDL7-EPIP treatment included *GLUTAMINE-DEPENDENT ASPARAGINE SYNTHASE* 1 (*ASN1*), *SMALL AUXIN UP RNA* (*SAUR*) and *INDOLEACETIC ACID-INDUCED PROTEIN 29* (*IAA29*) (Table 2). Since SAURs have been shown to be regulated by the basic helix–loop–helix (bHLH) transcription factor PHYTOCHROME INTERACTING FACTOR 4 (PIF4; Franklin *et al.*, 2011), we compared the genes in Table 2 with a list of PIF4 target genes identified by ChIP sequencing (Oh *et al.*, 2012). Six of 13 genes up-regulated by log_2_>1.0 by IDL7 treatment were PIF4 targets. In contrast, only one of 39 genes down-regulated by log_2_< –1.5 was a PIF4 target. *PIF4* itself was moderately, but significantly induced by IDL7-EPIP treatment (Supplementary Dataset S1).

To gain a further overview of the genes regulated by IDL7-EPIP, a network analysis of the most down-regulated genes in the gene set (log_2_< –1) was performed using the STRING database (Szklarczyk *et al.*, 2015). It is a general assumption that genes involved in the same processes are co-regulated or interact in one way or another, also known as the ‘guilt-by-association’ principle (Saito *et al.*, 2008). As shown in Supplementary Fig. S4, a tight cluster of highly connected genes was found within our array, indicating that IDL7 is regulating this network. The cluster includes the ZFP genes *ZAT6*, *ZAT12*, *ZAT10/STZ*, and *SZF1*, the WRKY transcription factor genes *WRKY33*, *WRKY40*, and *WRKY53*, and *CML38*, *ERF5*, and *ERF6.*

### IDL7 suppresses the expression of *ZAT12* upon wounding

**Fig. 5.**
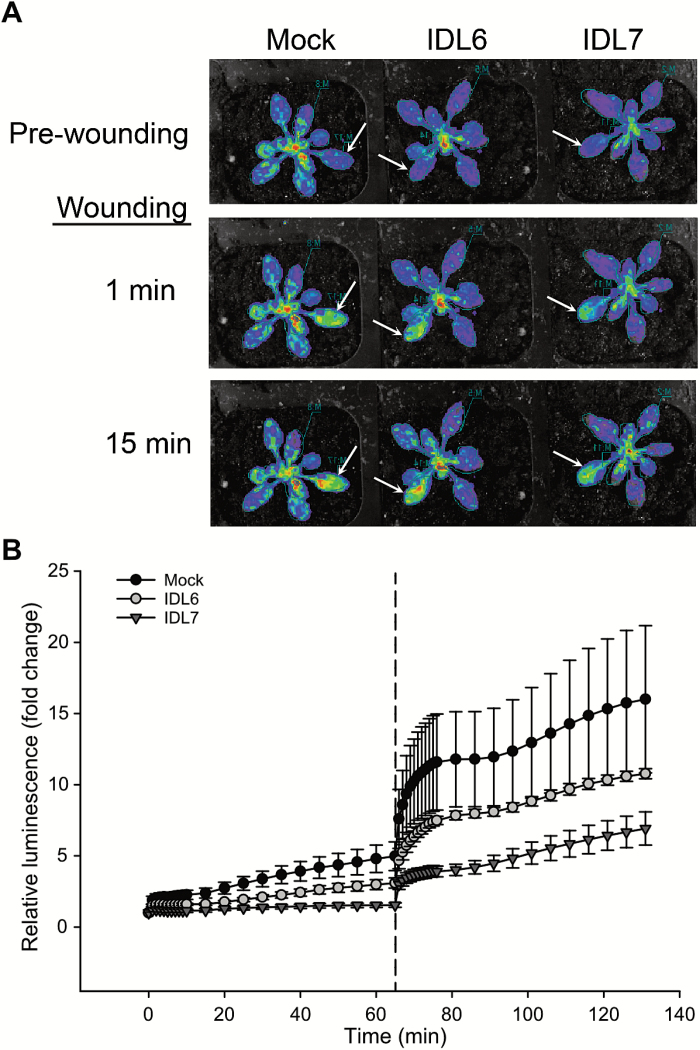
Suppression of the rapid wound-induced signal in *ZAT12::luc* plants by IDL6-EPIP and IDL7-EPIP peptides. Plants pre-treated with IDL6-EPIP, IDL7-EPIP, or MOCK_IDL7_ (100 nM) by spraying were simultaneously wounded in one leaf (marked with arrows), and changes in luminescence were immediately measured using the NightOwl *in vivo* imaging system. (A) Representative pictures are shown. Injured leaves are marked with arrows. (B) Quantification of the wound-induced signals in rosette leaves over the time course before and after wounding. Error bars indicate the SD between three biological replicates. (This figure is available in color at *JXB* online.)

One of the most down-regulated genes in our array was the ZFP gene *ZAT12*. *ZAT12* is highly responsive to different environmental conditions, and is rapidly induced after wounding and accumulation of ROS (Davletova *et al.*, 2005*a*; Miller *et al.*, 2009). Furthermore, in a study using the luciferase reporter gene, *ZAT12* expression was shown to be systemically induced at a rate up to 8.4 cm min^−1^ upon wounding (Miller *et al.*, 2009). We wanted to investigate the effect of treatment with IDL6-EPIP and IDL7-EPIP peptide prior to wounding on the expression of *ZAT12* using the *ZAT12::luciferase* reporter plants used in the study by Miller and co-workers (Miller *et al.*, 2009). Three plants per peptide were treated with luciferin and peptides (IDL6-EPIP, IDL7-EPIP, and MOCK_IDL7_), and 1 h later one rosette leaf per plant was wounded. Interestingly, as shown in Fig. 5, IDL7-EPIP was able to suppress spreading of the luciferase signal, verifying the negative effect of IDL7-EPIP on *ZAT12* expression. IDL6-EPIP was also able to suppress *ZAT12* expression, but the response was weaker. This trend was observed in three independent experiments.

### *IDL6* and *IDL7* are stress-responsive genes

**Fig. 6.**
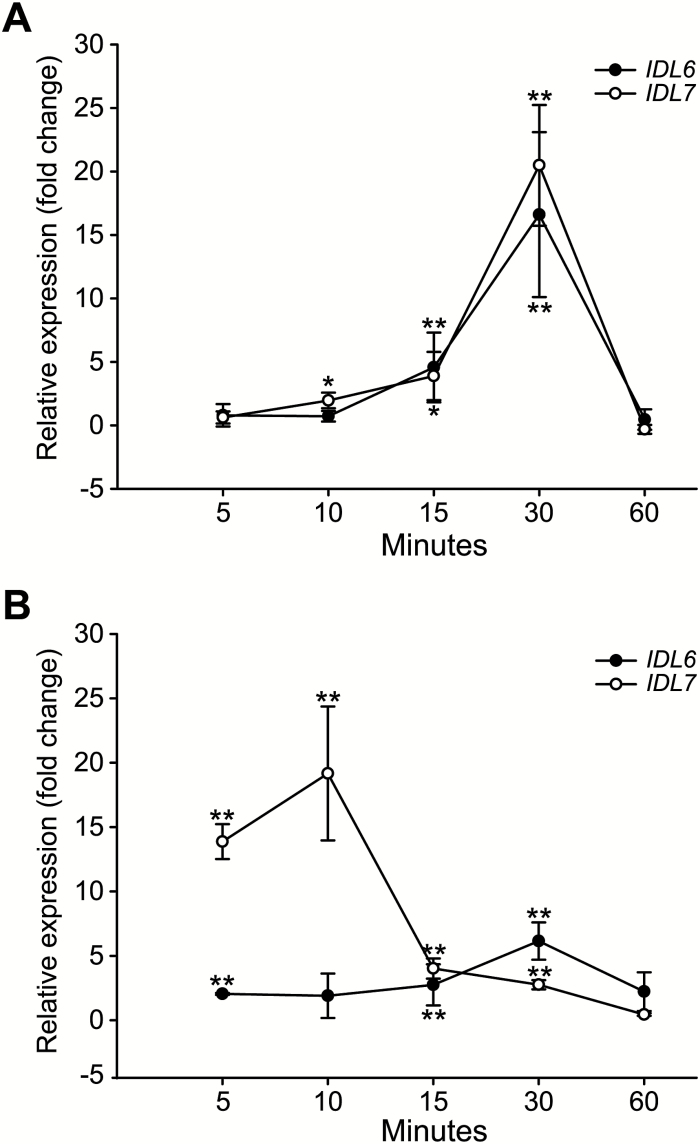
Time series analysis of *IDL6* and *IDL7* expression by qRT–PCR after flg22 treatment (A) and H_2_O_2_ treatment (B) compared with untreated tissue (*n*=4). Statistical differences (REST analysis: *P-value <0.05; **P-value <0.01) between the time point samples and control are indicated. Error bars indicate SDs.

*In silico* data indicate that *IDL6* and *IDL7* are moderately expressed during development, and that both genes are rapidly induced by stresses including exposure to *P. syringae* and the pathogen-associated elicitor flg22, salt, UV, wounding, and ROS (Vie *et al.*, 2015). To analyse the stress-induced expression of *IDL6* and *IDL7*, a time course experiment with Arabidopsis seedlings treated with flg22 or H_2_O_2_ was performed. Expression analyses using qRT–PCR analysis showed that the expression of both *IDL6* and *IDL7* was rapidly induced 10–15 min after flg22 treatment, and reached a peak after 30 min (Fig. 6A). The transcriptional response of *IDL7* to H_2_O_2_ treatment was even faster, reaching maximal levels after 10 min, whereas induction of *IDL6* expression was both weaker and slower (Fig. 6B).

T-DNA insertion lines of *IDL6* and *IDL7* were used for functional analysis. Loss of gene expression was verified using qRT–PCR. Two available T-DNA insertion lines were obtained for *IDL6*. Both *idl6* [SALK_074245 (*idl6-1*) and SALK_126026M (*idl6-2*)] lines contain a T-DNA insertion 1 bp upstream of the start codon, and low *IDL6* transcript levels were detected. However, *IDL6* expression in these mutant lines was not inducible with flg22 (Supplementary Fig. S5A), suggesting that *idl6-1* and *idl6-2* are knockdown mutants. Only one T-DNA insertion line was available for *IDL7*. *idl7* (WDL293-296) has the T-DNA inserted in the exon (after 188 bp), and is a null line with no detectable *IDL7* mRNA with or without induction of flg22 (Supplementary Fig. S5B). Neither *idl6* nor *idl7* plants displayed any observable phenotypical differences compared with wild-type control plants (Col-0) under normal growth conditions. Therefore, crosses between *idl6* and *idl7* plants were performed. However, neither *idl6-1 idl7* nor *idl6-2 idl7* double mutants displayed any obvious phenotypes during normal growth conditions, and no additive effect of the double mutation was found (results not shown). To investigate the potential change in gene expression in the mutant lines compared with Col-0, 2-week-old seedlings were treated with flg22 (100 nM) and samples harvested after 3 h, 1 h after the peak response to IDL7-EPIP. Surprisingly, the responses of *ZAT10* and *WRKY40* were slightly, but not significantly, reduced in all the mutant lines compared with Col-0 wild type (Supplementary Fig. S6).

### *idl7* loss-of-function lines are more tolerant to salt stress

The observed induction of *IDL6* and *IDL7* expression upon a broad range of environmental perturbations (Vie *et al.*, 2015) suggests that these genes are involved in modulating stress responses in plants. Seedlings of *idl6* and *idl7* were therefore exposed to different stresses and examined for seed germination, root growth, ion leakage, and pathogen growth. The following stresses were tested: elicitor treatment (100 nM flg22), pathogen infection (*P. syringae* DC3000 and *P. syringae* AvrRPM1), salinity stress (100 mM NaCl), and osmotic stress (300 mM mannitol).

No significant differences between wild-type plants and the mutant lines were observed after treatment with the pathogen-derived elicitor flg22 (Supplementary Fig. S7A). Similarly, no significant differences in growth of *P. syringae* were observed between wild-type plants and the mutant lines, neither with the virulent nor with the avirulent strain (Supplementary Fig. S7B, C). As shown in Supplementary Fig. S8A, both *idl6* and *idl7* lines showed significantly (*P*<0.01) increased tolerance to salinity stress, producing longer roots than the wild type under the same growth conditions. However, no additive effect was observed for the double loss-of-function mutant *idl6-2 idl7.* Complementation lines of *idl6* and *idl7* loss-of-function lines were used to verify the observed phenotype (Supplementary Fig. S8B). No significant differences in root growth were observed between *idl6* and *idl7* lines and the wild type grown in the presence of mannitol (Supplementary Fig. S8C). Hypocotyl elongation in the dark was also investigated, with no differences found between the wild type and the mutant lines (Supplementary Fig. S8D). Germination rates of *idl6* and *idl7* mutant lines on medium containing NaCl, mannitol, or abscisic acid (ABA) did not differ from the wild type (results not shown).

### IDL7 acts as a negative modulator of stress-induced ROS signalling

**Fig. 7.**
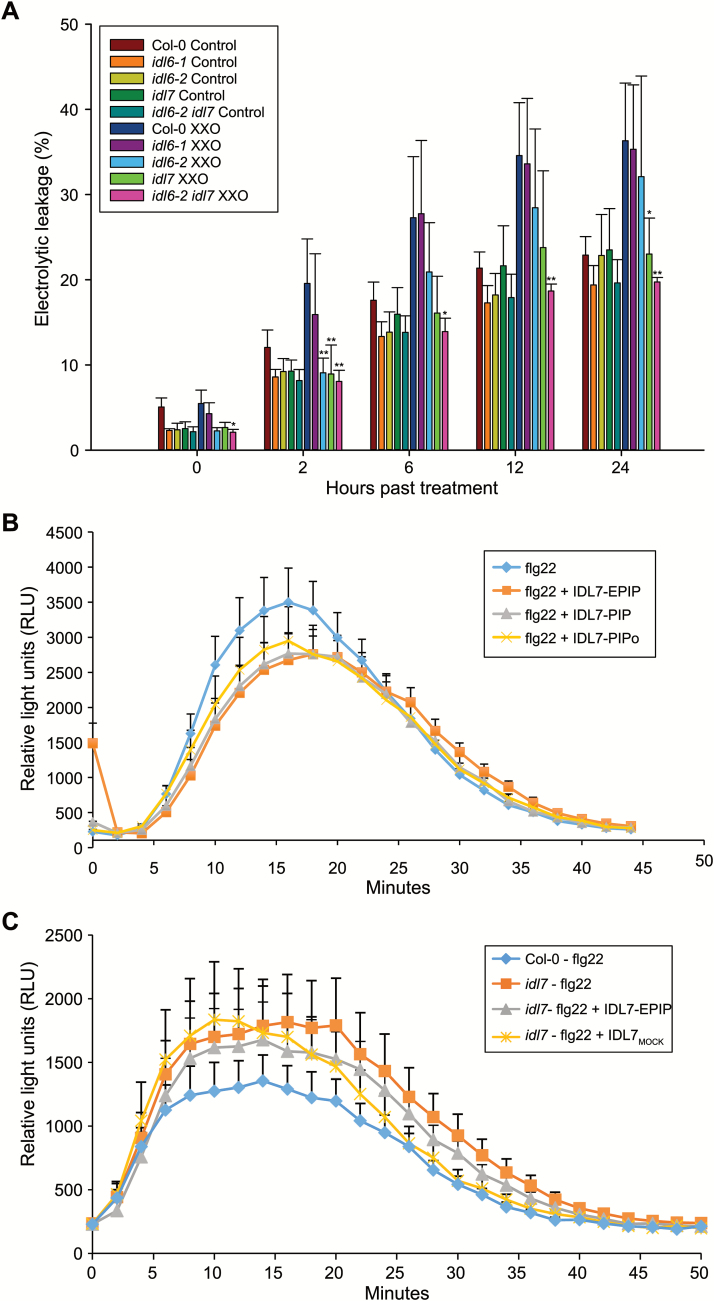
IDL6 and IDL7 are negative modulators of stress-induced ROS signalling. (A) Measurements of electrolytic leakage after treatments with the ROS-generating enzyme assay xanthine/xanthine oxidase (0.05 U) 2, 6, 12, and 24 h after treatment (*n*=4). Statistical differences for all experiments (Student’s *t*-test: *P-value <0.05, **P-value <0.01, ns indicates *P*-value >0.05) between the wild-type Col-0 and mutants are indicated. Error bars indicate SDs. (B, C) Modulation of flg22-induced oxidative burst by IDL7 peptide. Arabidopsis Col-0 wild-type (B) or *idl7* (C) leaf disks were exposed to flg22, IDL7-EPIP, IDL7-PIP, IDL7-PIPo, or MOCK_IDL7_ peptides (100 nM), either alone or in combination (flg22+IDL7, flg22+IDL7-PIP, flg22+IDL7-PIPo, and flg22+MOCK_IDL7_). Water was added as a control. ROS production measured as luminescence was monitored over time as relative light units (RLU). Error bars indicate the SE of *n*=12 replicates.

Since *IDL6* and especially *IDL7* expression was rapidly and strongly induced by H_2_O_2_, we investigated the response of *idl6* and *idl7* mutants to oxidative stress, using the xanthine/xanthine oxidase (X/XO) system. X/XO is an O_2_·^¯^-generating system that mimics and induces production of extracellular superoxide by NADPH oxidases, similar to O_3_. X/XO treatment results in cell death, which can be measured by electrolyte leakage (Overmyer *et al.*, 2005). X/XO treatments of *idl6-1*, *idl6-2*, *idl7*, and the *idl6-2 idl7* double mutant showed that *idl7*, but not *idl6*, was more tolerant to ROS, as indicated by less electrolyte leakage from *idl7* compared with the wild type and *idl6* over time (Fig. 7A).

In order to investigate further the role of the IDL7 peptide in ROS responses, we employed a luminol-based assay to measure ROS production in Arabidopsis leaf disks. In addition to IDL7-EPIP, we included two shorter synthetic peptides in this analysis; IDL7-PIP (see Fig. 3A) and the hydroxyprolinated IDL7-PIPo, as a recent publication on IDA has shown that a hydroxyprolinated 12 amino acid IDA-PIP peptide has higher biological activity than the IDA-EPIP peptide (Butenko *et al.*, 2014). As expected, treatment with flg22 peptide resulted in a rapid ROS burst, whereas no ROS production was observed in the control (Fig. 7B). Addition of IDL7 or MOCK_IDL7_ peptide alone did not lead to any ROS production (Supplementary Fig. S9). Interestingly, when IDL7-EPIP peptide was added together with flg22 peptide, the ROS burst was significantly (Student’s *t*-test, *P*<0.001) lower compared with flg22 peptide (Fig. 7B). Co-treatment with MOCK_IDL7_ peptide did not change the level of the ROS burst (Student’s *t*-test, *P*>0.05, Supplementary Fig. S9). However, treatments with the shorter IDL7-PIP peptide and the hydroxyprolinated IDL7-PIPo did not lead to a decrease in the ROS burst compared with IDL7-EPIP treatment (Fig. 7B). Flg22 treatment of *idl7* plants resulted in increased ROS production compared with the Col-0 wild type (Student’s *t*-test, *P*<0.001); this effect was partially suppressed by co-treatment with IDL7-EPIP (Student’s *t*-test, *P*<0.001, Fig. 7C). Thus, IDL7 peptide has an attenuating effect on the flg22-induced ROS burst.

## Discussion

In this study we describe two new putative peptide ligands belonging to the extended family of IDA and IDL peptides (Vie *et al.*, 2015) and show evidence suggesting that they act as negative modulators of ROS responses.

Small transcriptionally modified peptides are characterized by an N-terminal SP that directs the peptide out of the cell. Analyses of subcellular localization of IDA and CLE peptides indicate that they are localized to the extracellular space (Butenko *et al.*, 2003; Sharma *et al.*, 2003). Similar to the CLE peptides and IDA, IDL6 and IDL7 contain an N-terminal SP (Vie *et al.*, 2015). However, in addition to localization in the apoplast, a full-length fusion of IDL7::GFP was accumulated in a vesicular compartment (Fig. 1E, F). An IDL7_∆SP_::GFP fusion displayed cytosolic localization resembling GFP (Fig. 1A–D). Deletion of the four C-terminal amino acids of IDL7 resulted in a GFP fusion protein with apoplastic localization (Fig. 1G), which was confirmed through plasmolysis experiments (Fig. 1H). These results suggest that IDL7 is processed at both the N- and the C-terminus before or during the transport out of the cell. The EPIP/SGPS motif of all IDL subfamily members contains a C-terminal asparagine followed by between 4 and 13 poorly conserved residues (Vie *et al.*, 2015). Removal of Asn69 abolishes IDA activity in a ROS burst assay (Butenko *et al.*, 2014). Furthermore, IDA in complex with the ectodomain of its receptor HAESA (HAE) suggests that Asn69 constitutes the C-terminal residue of the mature IDA peptide *in planta* (Santiago *et al.*, 2016). Thus, it seems likely that C-terminal processing is a general and important feature of the maturation process of these peptides. Some of the C-terminal residues may confer specificity to the mature peptides. The N-terminus of the mature IDL6 and IDL7 peptides remains unresolved.

Our analyses indicate that the *IDL6* and *IDL7* promoters show high basal activity (Fig. 2); a similar observation was recently made by Wang *et al.* (2017) using an *IDL6* promoter:*GUS* construct. This is compensated by a high turnover rate for the transcripts. Thus, expression of a stable reporter gene transcript under control of the *IDL6* or *IDL7* promoter will lead to artificially high reporter levels (Supplementary Fig. S2). Alternatively, the binding site for a strong transcriptional repressor or another important regulatory unit, e.g. the 3'-untranslated region, could be missing in the promoter regions used in the *IDL6*_*pro*_*:GUS* and *IDL7*_*pro*_*:GUS* plants. A peptide ligand involved in stress signalling should be rapidly induced in order to ensure a quick response to biotic or abiotic challenges to the plant. However, the signal should also be quickly attenuated to ensure specificity and avoid runaway responses. Both *IDL6* and *IDL7* are transiently expressed upon H_2_O_2_ or flg22 treatment (Fig. 6; Wang *et al.*, 2017), in line with a role in early responses to these stresses.

Transcriptome analyses of seedlings treated with either IDL6-EPIP or IDL7-EPIP indicated that these peptides act as negative regulators of genes associated with early responses to stress in Arabidopsis seedlings (Figs 3, 4; Table 2; Supplementary Dataset S1, S2). Treatments with the synthetic IDL7-EPIP resulted in a striking transcriptome response, in which 75% of the significantly regulated genes were down-regulated. Several studies have proposed a ‘universal stress response transcriptome’ cluster in plants, which includes *IDL7*, *ZAT10*, *ZAT12*, *WRKY11*, *WRKY18*, *WRKY33*, *WRKY40*, and *CML38* (Kilian *et al.*, 2007; Ma and Bohnert, 2007; Walley *et al.*, 2007, Hahn *et al.*, 2013). In addition, network analysis of the genes most down-regulated by IDL7-EPIP (Supplementary Fig. S4) illustrates that these genes are highly co-expressed and probably involved in the same biological processes. Expression of *ZFP* and *WRKY* genes is highly responsive to a great variety of stresses; these transcription factor families have emerged as key regulators and integrators of ROS signalling in plants (Miller *et al.*, 2008). *ZAT12* has been described as a hyper-responsive gene to ROS accumulation, and is expressed both locally and systemically within minutes after increased ROS production (Miller *et al.*, 2009). ROS-induced expression of *ZAT12* was strongly reduced in response to treatment with IDL7-EPIP. Both the transcriptional changes trigged by IDL7-EPIP and the increased tolerance to ROS and salinity stress in *idl6* and *idl7* lines (Fig. 7A; Supplementary Fig. S7) suggest that IDL6 and IDL7 are negative modulators of stress responses in Arabidopsis.

Pathogen attack or environmental perturbations such as increased salinity induce a large network of different pathways containing both positive and negative regulatory components, where negative regulators may be important factors preventing excessive and unrestricted activation of defence mechanisms. Activation of the plant defence system has been shown to have a major impact on plant growth and fitness. In line with thi0s, overexpression of *ZAT7* leads to severe growth defects (Ciftci-Yilmaz *et al.*, 2007). Mutants for the MAP kinase MPK4 display severe growth retardation, possibly through overaccumulation of the defence-related phytohormone salicylic acid (SA) and H_2_O_2_ (Petersen *et al.*, 2000; Ichimura *et al.*, 2006; Suarez-Rodriguez *et al.*, 2007), and overexpression of *DREB2A*, a positive regulator of stress tolerance, showed reduced growth and dwarfism similar to *mpk4* mutants (Sakuma *et al.*, 2006).

Expression analyses of *IDL6* and *IDL7* show that these genes are responsive to a wide variety of biotic and abiotic stresses (Fig. 6; Vie *et al.*, 2015). However, both the single and double loss-of-function mutants displayed minor phenotypes in only a subset of the stress assays performed during this study. There are several possible explanations for the lack of phenotypes. One possibility is that IDL6 and IDL7 are part of a complex signalling network with redundant pathways, and that altered expression of *IDL6* and *IDL7* is not enough to disrupt this network noticeably. The gene expression of *ZAT10* and *WRKY40* was not different in the mutant lines compared with Col-0 after flg22 (Supplementary Fig. S6), possibly reflecting the lack of phenotypes observed in our stress assays. It is possible that IDL6 and IDL7 have multiple roles in tolerance to different stresses, and that our functional characterizations have not included all of the specific stresses linked to IDL6 and IDL7. In a recent study, *IDL6*-overexpressing plants were found to exhibit reduced resistance to *P. syringae* pv. *tomato* DC3000, whereas *IDL6* dsRNA-silenced plants showed increased resistance (Wang *et al.*, 2017). There are major differences between the two studies, with regard both to the growth stage of the plants (1 versus 5 weeks) and to the nature of the knockdown mutants (T-DNA versus dsRNA), which could explain the different outcomes.

The fact that treatments with X/XO and NaCl (Fig. 7; Supplementary Fig. S7) led to a phenotype in *idl7* lines is supported by the fact that a large fraction of the genes down-regulated by IDL7-EPIP treatment are associated with these stresses. WRKYs and ZFPs have been linked to oxidative and salinity stress (Rizhsky *et al.*, 2004; Davletova *et al.*, 2005*a*, *b*; Ciftci-Yilmaz *et al.*, 2007; Jiang and Deyholos, 2009; Chen *et al.*, 2010). Overexpression of *ZAT7* and *ZAT12* has been reported to increase tolerance to saline and oxidative stress (Rizhsky *et al.*, 2004; Davletova *et al.*, 2005*b*; Ciftci-Yilmaz *et al.*, 2007). The increased tolerance to salinity stress in the *idl6* and *idl7* loss-of-function lines are in line with these data.

In spite of its similarity to IDL7, IDL6-EPIP displayed a far weaker effect on transcriptional changes compared with IDL7-EPIP, indicating that IDL6 has lower biological activity than IDL7. Alternatively, IDL6 and IDL7 might have different roles in stress regulation, mediated through differences in expression patterns, receptor availability, and/or peptide modifications, or that the peptide is active at different growth stages from those investigated in this study. It is worth noting that IDL6-EPIP differs in four amino acids in comparison with IDL7-EPIP, where IDL7-EPIP contains one charged amino acid (arginine versus valine) more than IDL6-EPIP in front of the SGPS motif (Fig. 3A). It is also likely that many of the transcriptional targets of the two peptides are expressed at their basal, non-induced levels during normal growth conditions, explaining the few regulated genes for IDL6 and the moderate response for IDL7.

Several lines of evidence point toward a role for IDL7, and possibly IDL6, as a modulator of ROS signalling. The transcriptional response of *IDL7* to H_2_O_2_ treatment is rapid (Fig. 6B). The temporal difference in *IDL7* induction by H_2_O_2_ and flg22 could correspond to the time needed for flg22 to induce a ROS burst (Gómez-Gómez *et al.*, 1999; Fig. 7B). Application of exogenous IDL7-EPIP (and to a minor extent IDL6-EPIP) peptide blocked the rapid induction of *ZAT12* expression after wounding (Fig. 5), which has been shown to be mediated by ROS (Miller *et al.*, 2009). *idl7* and *idl6-2 idl7* double mutants displayed decreased sensitivity to ROS produced by X/XO (Fig. 7A). Importantly, the IDL7 peptides attenuated the flg22-induced ROS burst and partially rescued the enhanced ROS production in the *idl7* background (Fig. 7B, C). The fact that the synthetic IDL7 peptide fails to rescue the *idl7* ROS phenotype completely suggests that it is not identical to the mature IDL7 peptide with regard to length and/or post-translational modification. However, counterintuitively, both the shorter IDL7-PIP and the hydroxyprolinated IDL7-PIPo resulted in a similar response to the longer IDL7-EPIP peptide (Fig. 7B). At this point, we cannot draw any conclusions regarding the length of the biological peptide. It is possible that the bioactive IDL7 peptide is longer than IDA, but still contains modifications, or that the sensitivity of our assays is not high enough to detect the possible stronger effects of the more active peptide.

The receptor(s) for the IDL7 peptide are unknown; however, IDL7 perception is likely to involve LRR-RLKs that phosphorylate downstream targets. Binding of IDA to its receptors HAE or HAESA-LIKE2 (HSL2) activates a mitogen-activated protein (MAP) kinase cascade that results in the phosphorylation of transcription factors (Cho *et al.*, 2008; Patharkar and Walker, 2015). Furthermore, the effect of IDL6 on resistance to *P. syringae* and pectin degradation was found to be dependent on HAE and HSL2 (Wang *et al.*, 2017). A pathway possibly involving HAE/HSL2 could act downstream of IDL7 to down-regulate genes of the ‘universal stress response transcriptome’, through either activation of a transcriptional repressor, or inactivation of a transcriptional activator. However, this scenario does not explain the attenuating effect of the IDL7 peptides on ROS production when added together with flg22. Alternatively, downstream targets of IDL7 signalling could be involved in regulation of ROS levels. An attractive group of target candidates would be NADPH oxidases, especially RBOHD. In addition to calcium binding, RBOH activity is regulated by phosphorylation by several classes of protein kinases (Baxter *et al.*, 2014; Kimura *et al.*, 2017). IDL7 signalling could regulate RBOH phosphorylation status, either by inactivating one or more of these kinases, or by activating a phosphatase with specificity toward RBOHs. Instead of inhibiting ROS production, IDL7 signalling could potentially activate ROS scavenging enzymes.

What could be the role of IDL6/IDL7-regulated ROS attenuation? IDL7 (and possibly IDL6) may act in a negative feedback loop terminating the fast phase of the biphasic ROS burst. Mild salt treatment induces a biphasic RBOHD-dependent ROS burst similar to the one induced by flg22 (Xie *et al.*, 2011), and the *rbohd rbohf* double mutant displays increased salt sensitivity (Ma *et al.*, 2012; Ben Rejeb *et al.*, 2015). The strong negative effect of IDL6/IDL7 treatment on wound-induced *ZAT12* expression also suggests that these peptides might be involved in attenuation of the autopropagating ‘ROS wave’ leading to systemic acquired acclimation (Baxter *et al.*, 2014; Gilroy *et al.*, 2014). Such attenuation could be important for specificity of the signal, as well as avoiding runaway responses.

## Supplementary data

Supplementary data are available at *JXB* online.

Fig. S1. Overview of the constructs used for localization studies.

Fig. S2. Distribution of *GUS* mRNA directed by the *IDL6* and *IDL7* promoters in 10-day-old seedlings.

Fig. S3. Venn diagram of IDL6- and IDL7-responsive genes.

Fig. S4. Network analysis of genes down-regulated by IDL7-EPIP treatment.

Fig. S5. Verification of T-DNA insertion lines.

Fig. S6. Expression of IDL7-responsive genes in *idl6* and *idl7* mutant backgrounds.

Fig. S7. Growth arrest phenotype and susceptibility of *idl6* and *idl7* mutants to the phytopathogen *Pseudomonas syringae*.

Fig. S8. Growth arrest phenotype of *idl6* and *idl7* mutants to abiotic stress.

Fig. S9. Modulation of flg22-induced ROS burst by IDL7-EPIP and MOCK_IDL7_.

Table S1. List of PCR primers used in this study.

Dataset S1. Genes significantly regulated (*P*<0.05) 2 h after treatment with IDL7-EPIP.

Dataset S2. Genes significantly regulated (*P*<0.05) 2 h after treatment with IDL6-EPIP.

Dataset S3. Raw data from GO analysis of significantly down-regulated (*P*<0.05) genes by IDL7-EPIP treatments.

## Supplementary Material

Supplementary Figures S1-S9 and Table S1

Supplementary Datasets S1-S3
