# Supplementary material for: The IDA-LIKE peptides IDL6 and IDL7 are negative modulators of stress responses in Arabidopsis thaliana
Source: J Exp Bot. 2017 Jun 6;68(13):3557–71. doi: 10.1093/jxb/erx168 (PMC5853212; doi:10.1093/jxb/erx168)
Supplement: Supplementary Figures S1-S9 and Table S1 [file erx168_suppl_supplementary_figuress1-s9_tables1.pdf]

Supplementary Figure S1

A

1 MAINRSLLLI LLFISVSLST ARILPGEFVP VIFSGEIPPV SKSAVVGCGG

51 EQETKTEYSS FVPEVVAGRF GSLVLNALPK GSRPGSGPSK KTNDVKT

B

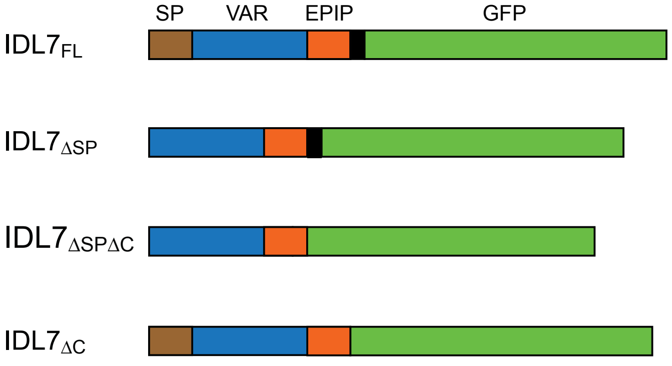

Supplementary Figure S1.
Overview of the construct used for localisation studies. (A) Protein sequence of *A. thaliana* IDL7. Green letters; signal peptide (SP), blue letters; variable region (VAR), orange letters; peptide motif (EPIP/SGPS), black letters; C-terminal. (B) Schematic presentation of IDL7-GFP fusions used in localisation studies. Colouring is the same as in (A).

Supplementary Figure S2

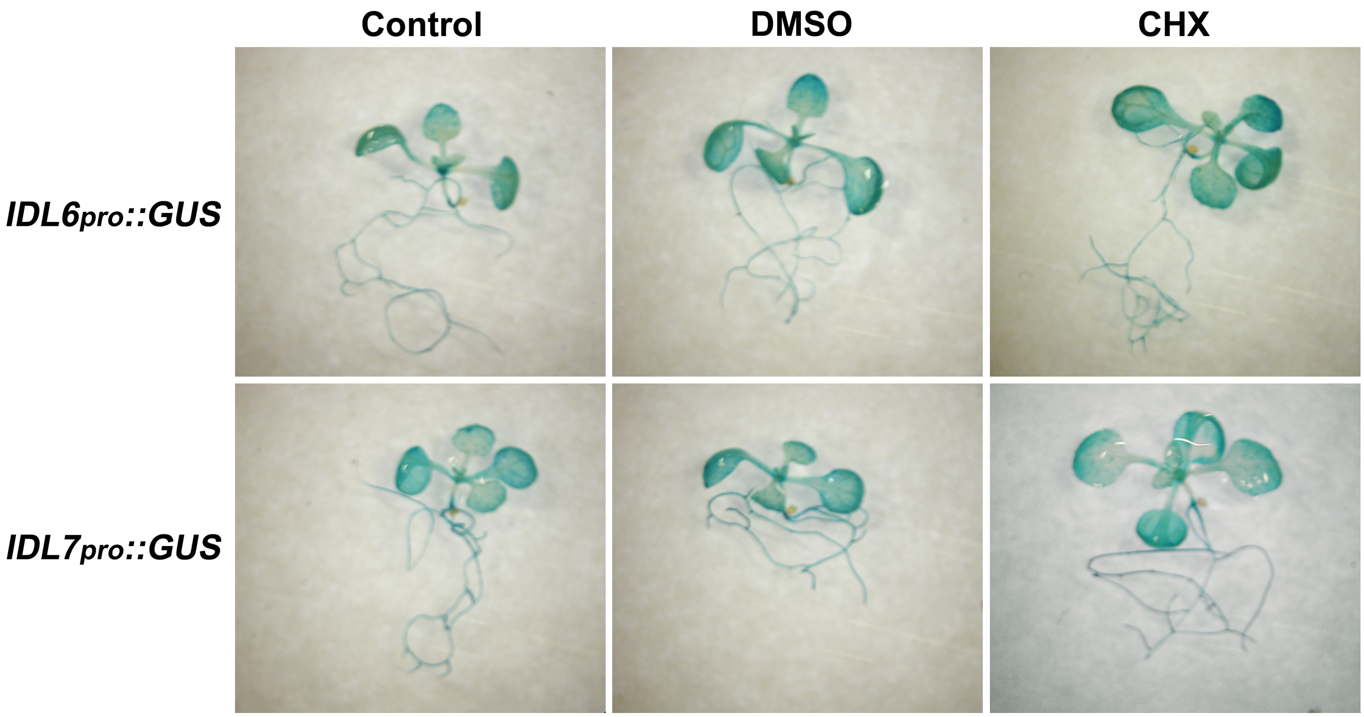

Supplementary Figure S2.
Distribution of *GUS* mRNA directed by the *IDL6* and *IDL7* promoters in ten days-old seedlings. Untreated plants (Control), plants treated with mock solution (0.1% DMSO, 3 hours), and plants treated with CHX (10 µg/ml, 3 hours).

Supplementary Figure S3

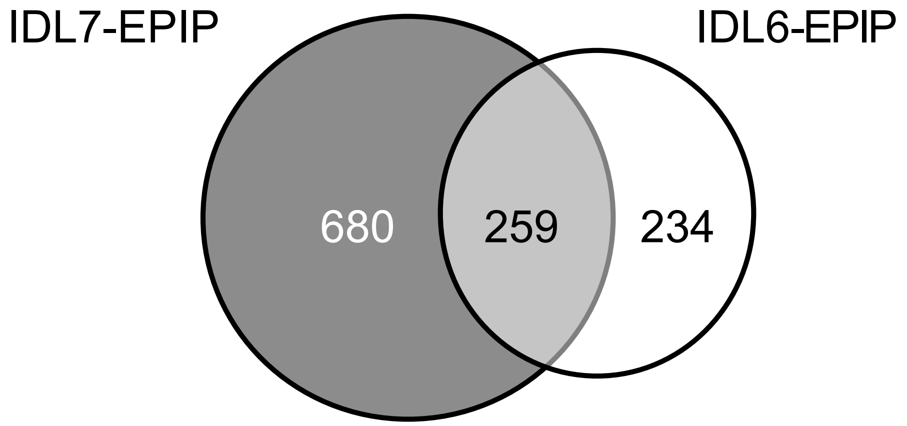

Supplementary Figure S3.
Venn diagram of IDL6- and IDL7-responsive genes. Genes significantly regulated by IDL6-EPIP ($p<0.1$) or IDL7-EPIP ($p<0.05$) peptide treatment were compared. Numbers indicate genes that have shared or unique regulation between the two datasets.

Supplementary Figure S4

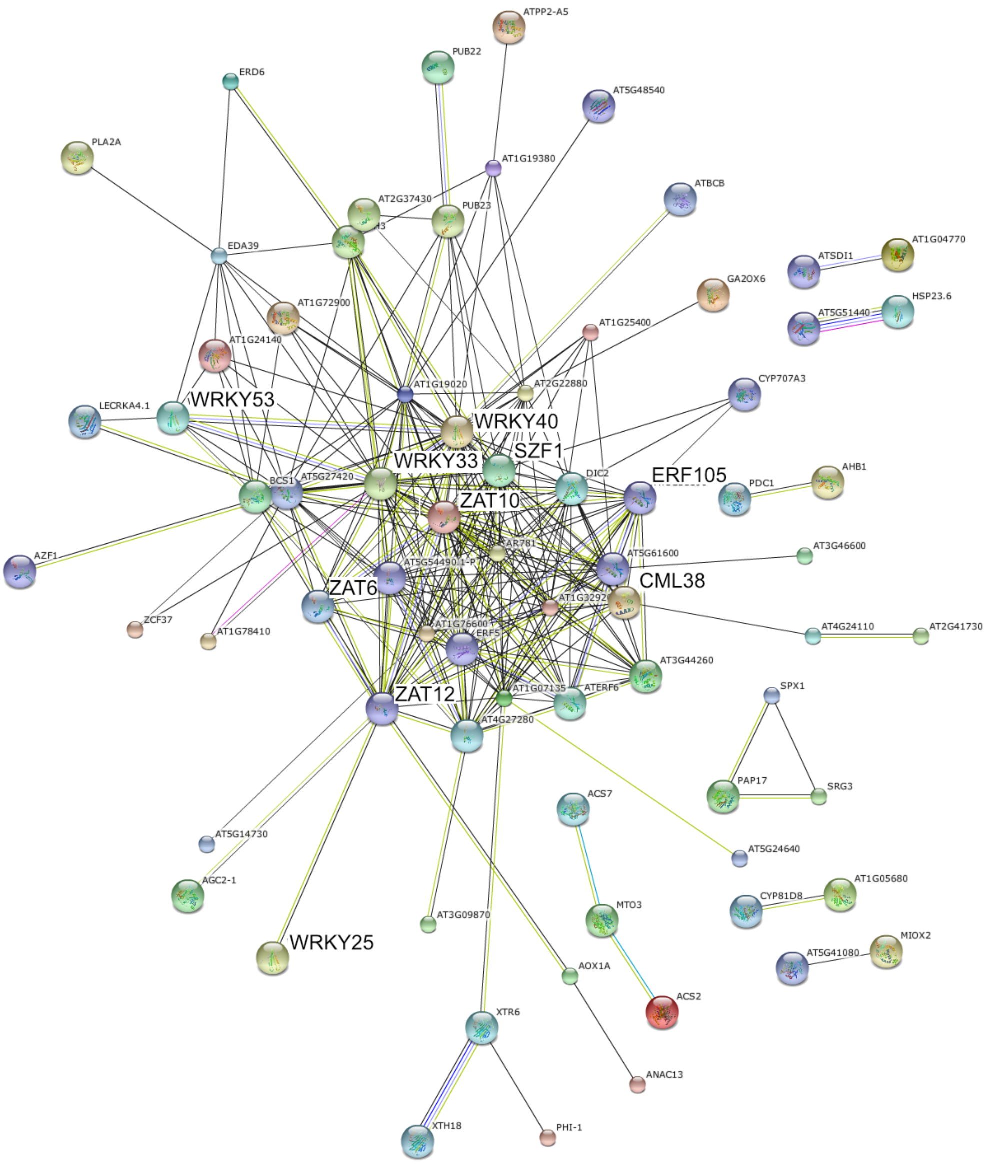

Supplementary Figure S4.
Network analysis of genes down-regulated by IDL7 peptide treatment. The web-based tool STRING (Szklarczyk *et al.*, 2015) was used on a filtered dataset (log2<-1; confidence level 0.700). The STRING database assembles information about co-occurrence, co-expression, databases, and text mining, which are also marked with different line colours: Green – neighbourhood; Red – gene fusion; Blue – co-occurrence; Black – coexpression; Pink – experiments; Turquoise – databases; Light green – text mining; Light blue – homology. Proteins with known structures are indicated with larger nodes. Disconnected nodes are removed. Discussed genes are written in large letters.

Supplementary Figure S5

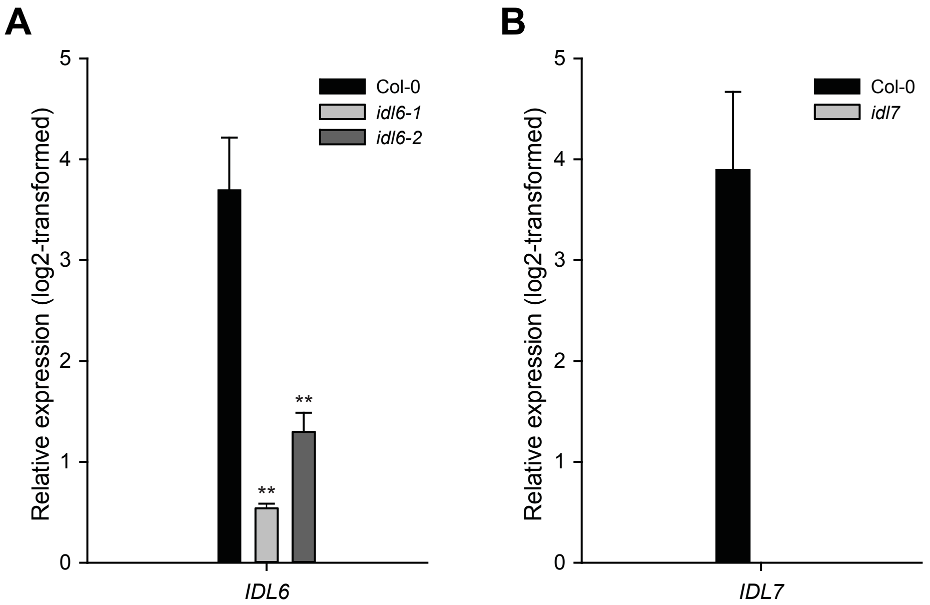

Supplementary Figure S5.
Verification of T-DNA insertion lines. T-DNA insertion lines for IDL6 and IDL7 were treated with 100 nM flg22 or a mock solution, and expression levels were analysed by qRT-PCR (n=3). No transcript was detected for the *idl7* mutant, while residual mRNA levels were detected in both *idl6-1* and *idl6-2* mutants. However, no increase in mRNA levels was detected upon flg22 treatment, indicating loss-of-function mutants for *IDL6*. Statistical differences (Rest analysis: *, p value<0.05; **, p value<0.01) between treated samples and control are indicated. Error bars indicate standard deviations.

Supplementary Figure S6

A

ZAT10						*WRKY40*					
	Col-0	*idl6-1*	*idl6-2*	*idl7*	*idl6-2 idl7*		Col-0	*idl6-1*	*idl6-2*	*idl7*	*idl6-2 idl7*
Col-0	1					Col-0	1				
idl6-1	0.88	1				*idl6-1*	0.92	1			
idl6-2	1.24	1.41	1			*idl6-2*	1.38	1.50	1		
idl7	0.92	1.04	0.74	1		*idl7*	0.82	0.89	0.59	1	
idl6-2 idl7	1.01	1.15	0.81	1.10	1	*idl6-2 idl7*	1.05	1.15	0.76	1.29	1

B

ZAT10						*WRKY40*					
	Col-0	*idl6-1*	*idl6-2*	*idl7*	*idl6-2 idl7*		Col-0	*idl6-1*	*idl6-2*	*idl7*	*idl6-2 idl7*
Col-0	1					Col-0	1				
idl6-1	1.39	1				*idl6-1*	1.12	1			
idl6-2	1.54*	1.11	1			*idl6-2*	1.32	1.18	1		
idl7	1.45	1.03	0.94	1		*idl7*	1.06	0.94	0.80	1	
idl6-2 idl7	1.47	1.05	0.95	1.02	1	*idl6-2 idl7*	1.35	1.20	1.02	1.27	1

Supplementary Figure S6.
Expression of IDL7-responsive genes in *idl6* and *idl7* mutant backgrounds. The expression of *ZAT10* and *WRKY40* after flg22 treatment were analysed in the *idl6* and *idl7* mutant lines by qRT-PCR. One-way ANOVA were used to compare the expression of the genes in all lines after A) control treatment and B) flg22 (100 nM) treatment, n=4. The values shows the relative expression of *ZAT10* or *WRKY40* between the different lines, as indicated by the matrix. Asterisks indicate significant differences ($p > 0.05$).

Supplementary Figure S7

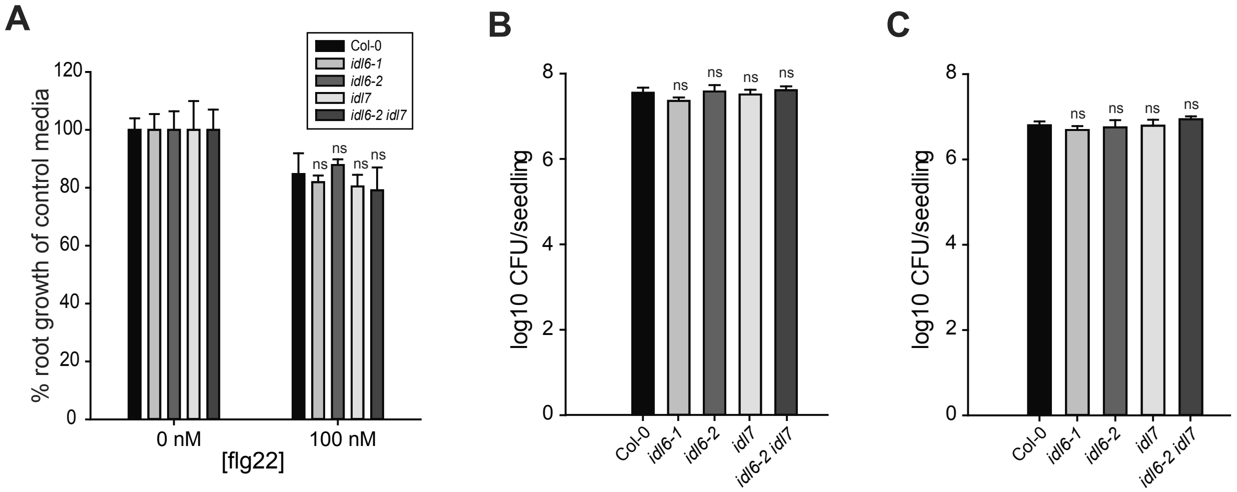

Supplementary Figure S7.
Growth arrest phenotype and susceptibility of *idl6* and *idl7* mutants to the phytopathogen *Pseudomonas syringae*. (A) Root lengths of wild-type and *idl6* and *idl7* loss-of-function mutants and wild-type were measured after growth on agar plates without or with Flg22 (100 nM) for ten days. n=48. (B) Pathogen susceptibility were investigated by infecting seven-day old seedlings of loss-of-function lines and wild-type grown in liquid half-strength MS media with ≈1.25×10^5 CFU/ml *P. syringae* DC3000 (B), and *P. syringae* AvrRPM1 (C). Bacterial growth was determined by counting cell forming units (CFU) after extraction and plating on agar plates three days after infection (n=3, 3 seedlings per replica). Statistical differences for all experiments (Student's t-test: ns indicates p-value>0.05) between the wild-type Col-0 and mutants are indicated. Error bars indicate standard deviations.

Supplementary Figure S8

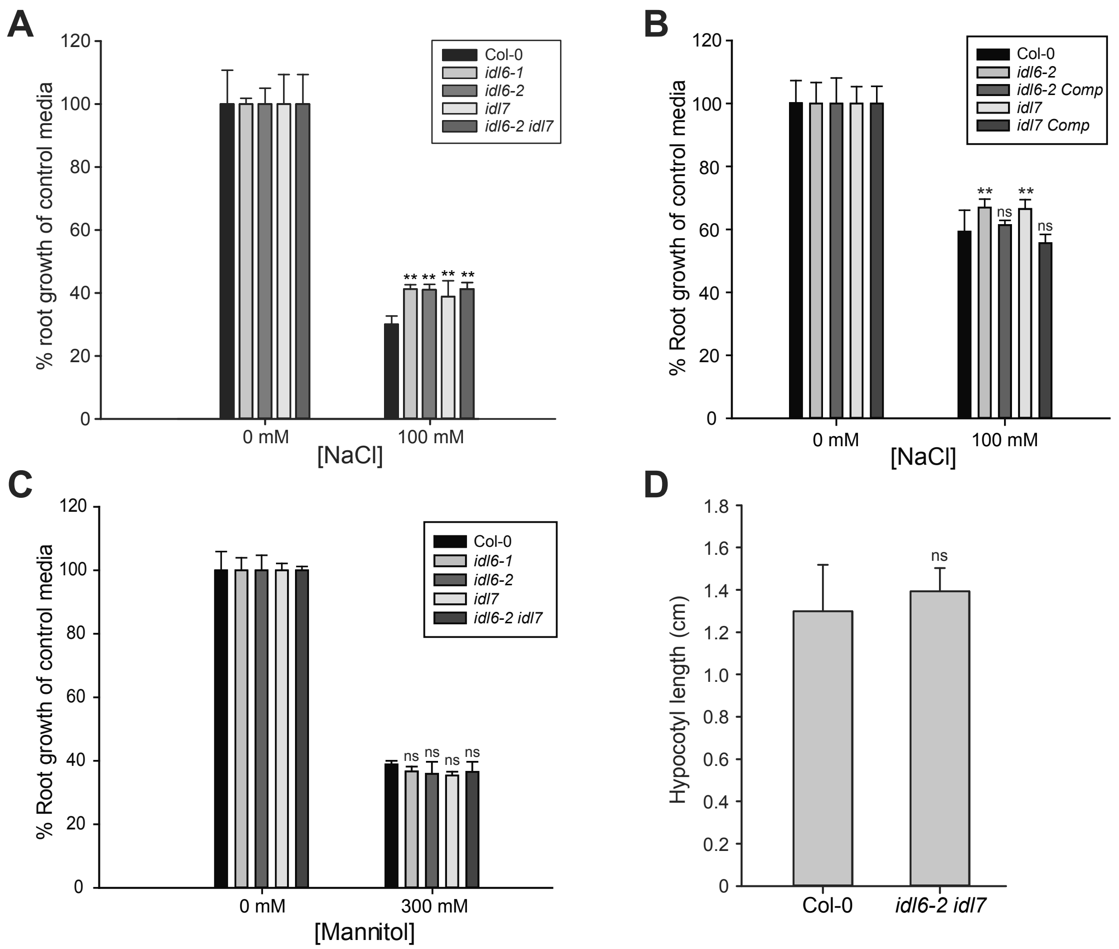

Supplementary Figure S8.

Growth arrest phenotype of *IDL6* and *IDL7* mutants. (A) Root lengths of wild-type and loss-of-function mutants and wild-type were measured after growth on half-strength MS medium without NaCl (0 mM) or with NaCl (100 mM) for one week (n=48). (B) The observed phenotypes of the loss-of-function mutants were verified by complementation. Root lengths of loss-of-function lines, complementation lines and wild-type were measured and compared after growth on half-strength MS without NaCl (0 mM NaCl) and with NaCl (100 mM NaCl) after one week growth. n=48. (C) Root lengths of loss-of-function mutants, plants over-expressing *IDL6* and *IDL7* and wild-type were measured after growth on half-strength MS medium without mannitol (0 mM mannitol) or with (300 mM mannitol) for one week (n=48). (D) Hypocotyl lengths of loss-of-function lines, overexpression lines and wild-type were measured after one week growth in the dark. n=36. Statistical differences for all experiments (Student's t-test: *, p-value<0.05, ** p-value<0.01, ns indicates p-value>0.05) are indicated. Error bars indicate standard deviations.

Supplementary Figure S9

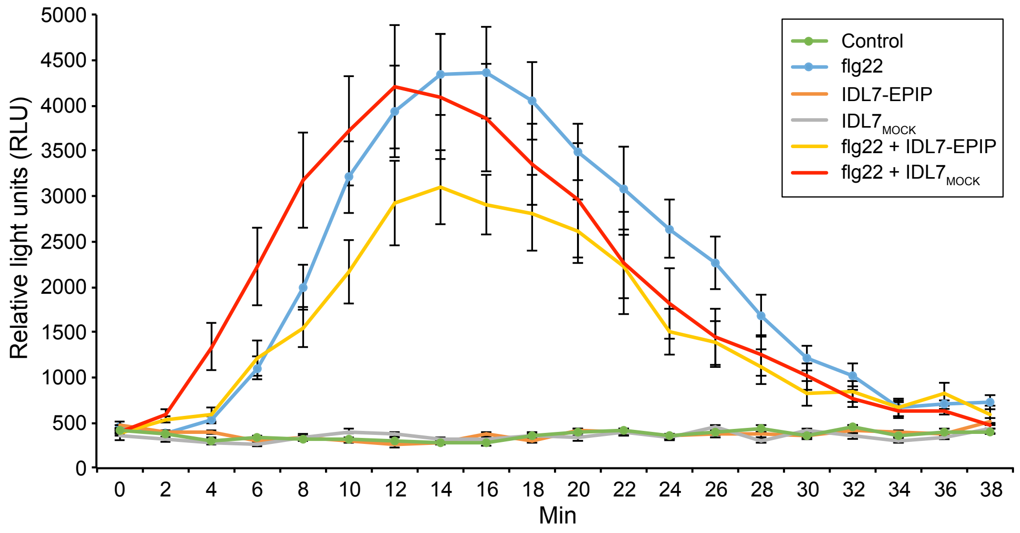

Supplementary Figure S9.
Modulation of flg22-induced oxidative burst by IDL7 peptide. Arabidopsis Col-0 wild-type leaf disks were exposed to flg22, IDL7 or $MOCK_{IDL7}$ peptides (100 nM), either alone or in combination (flg22 + IDL7 and flg22 + $MOCK_{IDL7}$). Water was added as a control. ROS production measured as luminescence was monitored over time as relative light units (RLU). Error bars indicate SE of n = 12 replicates. The experiment was repeated three times with similar results.

Supplementary Table S1. List of primers used in this study.

Primer name	Sequence	Used for
qCyp71A13F	TAAAGAGGTGCTTCGGTTGC	qRT-PCR, negative RT-control
qCyp71A13R	TATCGCAGTGTCTCGTTGGA	qRT-PCR, negative RT-control
qIDL6F	TGCTTCGTTCTCAACAGCTAGG	qRT-PCR
qIDL6R	GAATATCCAGCCGTCAAGTGAT	qRT-PCR
qIDL7F	CCGGAGAGTTTGTTCCAGTCAT	qRT-PCR
qIDL7R	CGTTAGTTTTCTTGCTGGGTCC	qRT-PCR
qGUSF		qRT-PCR
qGUSR		qRT-PCR
qTIP41-likeF	GTGAAAACTGTTGGAGAGAAGCAA	qRT-PCR reference gene
qTIP41-likeR	TCAACTGGATACCCTTTCGCA	qRT-PCR reference gene
qWRKY33F1	GACATTCTTGACGACGGTTACA	qRT-PCR
qWRKY33R1	CGATGGTTGTGCACTTGTAGTA	qRT-PCR
qWRKY40F1	CTGACACTACCCTCGTTGTGAA	qRT-PCR
qWRKY40R1	ACAGCTTGGAGCACAAGCACAT	qRT-PCR
qZAT12F	AAGCAGTTTCATTCGTTCCAAG	qRT-PCR
qZAT12R	TTCTTCATCAATCCAGACGACA	qRT-PCR
qZAT10F	GGAGGAGATGATCATTCAACCT	qRT-PCR
qZAT10R	CTTGTTACAGATGGTGCAAACG	qRT-PCR
pIDL6attB1	GGGGACACGTTTGTACAAAAAAGCAGGCTTCTTGGTACCCTCAAGCT	Promoter and complementation lines
pIDL6attB2	GGGGACCACTTTGTACAAGAAAGCTGGGTCAGCTCCAATTCTAGCCAT	Promoter lines
pIDL7attB1	GGGGACACGTTTGTACAAAAAAGCAGGCTTCTAAAGTGATGGCACGACTT	Promoter and complementation lines
pIDL7attB2	GGGGACCACTTTGTACAAGAAAGCTGGGTCGTTAATCGCCATCTGTAA	Promoter lines
IDL6komp attB2	GGGGACCACTTTGTACAAGAAAGCTGGGTCATTTTAATTTCAGTAGTAAC	Complementation lines
IDL7komp attB2	GGGGACCACTTTGTACAAGAAAGCTGGGTAATTCAAATGGTATCCTTGACT	Complementation lines
IDL7DSPattB1	GGGGACAAGTTTGTACAAAAAAGCAGGCTTCAGGATCTTACCCGGAGAG	GFP lines
IDL7USattR	GGGGACCACTTTGTACAAGAAAGCTGGGTAAGTCTTGACGTCGTTAGT	GFP lines
SPIDL7attB1	GGGGACAAGTTTGTACAAAAAAGCAGGCTTCACCCACTTCATCATTTTACA	GFP lines
IDL7EPIPattB2	GGGGACCACTTTGTACAAGAAAGCTGGGTCGTTAGTTTTCTTGCTGGGTCC	GFP lines
IDL6-cdsFWD	GGGGACAAGTTTGTACAAAAAAGCAGGCTTTATGGCTAGAATTGGAGCT	Over-expression lines
IDL6-cdsREV	GGGGACCACTTTGTACAAGAAAGCTGGGTCCTAAGTCTTGACGTCGTT	Over-expression lines
IDL7attF	GGGGACAAGTTTGTACAAAAAAGCAGGCTTGATGGCGATTAACAGATCT	Over-expression lines
IDL7attMSR	GGGGACCACTTTGTACAAGAAAGCTGGGTCCTAAGTCTTGACGTCGTTAG	Over-expression lines

IDL6KO123F	TGGTCTTTTCAGATAGGTGTGT	Verification of IDL6 T-DNA insertion line, gene specific
KO_209	AATCAAAGCTCCAATTCTAGCC	Verification of IDL6 T-DNA insertion line, gene specific
IDL7KO1F	CGTTAGTTTTCTTGCTGGGTCC	Verification of IDL7 T-DNA insertion line, gene specific, qRT-PCR
KO_210	CCGGAGAGTTTGTTCCAGTCAT	Verification of IDL7 T-DNA insertion line, gene specific, qRT-PCR
LBN	CGGAACCACCATCAAACAGGAT	Verification of T-DNA insert SALK lines, T-DNA specific
p745	AACGTCCGCAATGTGTTATTAAGTTGTC	Verification of T-DNA insert Wisconsin lines, T-DNA speceific
